# Association between bilirubin and risk of Non-Alcoholic Fatty Liver Disease based on a prospective cohort study

**DOI:** 10.1038/srep31006

**Published:** 2016-08-03

**Authors:** Jianbo Tian, Rong Zhong, Cheng Liu, Yuhan Tang, Jing Gong, Jiang Chang, Jiao Lou, Juntao Ke, Jiaoyuan Li, Yi Zhang, Yang Yang, Ying Zhu, Yajie Gong, Yanyan Xu, Peiyi Liu, Xiao Yu, Lin Xiao, Min Du, Ling Yang, Jing Yuan, Youjie Wang, Weihong Chen, Sheng Wei, Yuan Liang, Xiaomin Zhang, Meian He, Tangchun Wu, Ping Yao, Xiaoping Miao

**Affiliations:** 1 Department of Epidemiology and Biostatistics and the Ministry of Education (MOE) Key Lab of Environment and Health, School of Public Health, Tongji Medical College, Huazhong University of Science and Technology, 13 Hangkong Rd, Wuhan, Hubei, China; 2 Department of Nutrition and Food Hygiene, Hubei Key Laboratory of Food Nutrition and Safety and the Ministry of Education (MOE) Key Lab of Environment and Health, School of Public Health, Tongji Medical College, Huazhong University of Science and Technology, 13 Hangkong Rd, Wuhan, 430030, Hubei, China; 3 Division of Gastroenterology, Department of Internal Medicine, Union Hospital, Tongji Medical College, Huazhong University of Science and Technology, Wuhan, Hubei Province, China; 4 Institute of Occupational Medicine and the Ministry of Education (MOE) Key Lab of Environment and Health, School of Public Health, Tongji Medical College, Huazhong University of Science and Technology, 13 Hangkong Rd, Wuhan, 430030, Hubei, China; 5 Department of Maternal and Child Health, School of Public Health, Tongji Medical College, Huazhong University of Science and Technology, 13 Hangkong Rd, Wuhan, 430030, China

## Abstract

The study aimed to assess the association between total, direct, and indirect bilirubin and nonalcoholic fatty live disease (NAFLD) risk given its high prevalence and serious clinical prognosis. Among 27,009 subjects who participated in a healthy screening program from the Dongfeng-Tongji cohort study in 2008, 8189 eligible subjects (aged 35–86 years; males, 43.95%) were ultimately enrolled. The incidence rates of NAFLD in 2013 were compared with respect to baseline bilirubin levels among subjects free of NAFLD, and the effect sizes were estimated by logistic regression analysis. During 5 years follow-up, we observed 1956 cases of newly developed NAFLD with the overall incidence of 23.88%. Direct bilirubin was presented to inversely associate with NAFLD risk. Compared with quartile 1 of direct bilirubin, the multivariable-adjusted ORs (95% CIs) for NAFLD of quartile 2 to 4 were 1.104 (0.867–1.187), 0.843 (0.719–0.989), and 0.768 (0.652–0.905), respectively, *P* for trend 0.002). Similarly, inverse effects of direct bilirubin on NAFLD incidence were also observed when stratified by sex and BMI. However, no significant associations were found between total, and indirect bilirubin and NAFLD risk. Direct bilirubin reduced NAFLD risk independent of possible confounders among middle-aged and elderly Chinese population, probably based on the endogenous antioxidation of bilirubin.

Nonacoholic fatty live disease(NAFLD), a common clinicopathological disorder characterized by excessive lipids deposition in the hepatocytes, encompasses a wide range of histological spectrums, ranging from benign steatosis to inflammatory nonalcoholic steatohepatitis (NASH)^1^,^2^. NAFLD has become the most common cause of chronic liver disease worldwide with prevalence approximately ranging from 9% to 36.9% among general population and rising up to 30–50% in diabetes and 80–90% in obesity^3^,^4^. In China, with the steadily increasing pandemic of obesity and diabetes, the prevalence of NAFLD which approximately doubled in the past decade has reached to 15–20% in affluent regions of China^5^,^6^,^7^. In addition, the rapidly expanding body of clinical evidence supports NAFLD as a multisystem disease which may aggravate morbidity and mortality from cirrhosis, liver failure, hepatocellular carcinoma, and cardiovascular disease by disturbing hepatic structure and function^1^,^8^,^9^,^10^. Furthermore, it has also been shown that NAFLD is increasingly emerging as most common indication for liver transplant^11^. Therefore, it is critically important to identify the risk of NAFLD considering its high prevalence, serious clinical prognosis, and enormous public healthy challenges.

Serum bilirubin, the end product of haem metabolism, has been found to possess potential antagonizing oxidative stress and inflammatory properties by acting as antioxidant and cytoprotectant *in vitro* and *in vivo*^12^,^13^. Besides, there has been accumulating evidence frequently documenting not only oxidative stress, but insulin resistance was considered to be major triggers to NAFLD pathogenesis and progression^14^,^15^. Furthermore, growing evidence have indicated that bilirubin not only was thought to be an emerging biomarker of chronic disease resistance but also conferred a decreased risk of some diseases related to oxidative stress, including diabetes, metabolic syndrome, coronary artery disease and atherosclerosis^16^,^17^,^18^,^19^. Meanwhile, NAFLD is frequently demonstrated to strikingly associate with the risk of metabolic syndrome, type 2 diabetes, and cardiovascular diseases independent of other classical risk factors^20^,^21^,^22^. Therefore, a straightforward hypothesis has been proposed that bilirubin may contribute to protection against NAFLD risk, probably based on the antioxidant effects of bilirubin.

So far, several previous studies have been performed to examine the association between bilirubin levels and the risk of NAFLD^23^,^24^,^25^,^26^. However, most of these studies were performed based on a relatively small sample sizes. Moreover, certain limitations of evaluating causal relation and reliability of the results have been posed when interpreting the association by the cross-sectional or case-control study. In addition, the majority of these studies only accessed the effect of one type bilirubin on risk of NAFLD but not all subtypes.

Given aforementioned limitations, a prospective cohort study has been performed to evaluate the independent correlation between serum bilirubin levels (direct, indirect, total) and NAFLD risk among a large-scale middle aged and elderly Chinese population enrolled from Dongfeng-Tongji cohort study. Clarification of the association may help to explain the underlying mechanisms and more importantly, may be of remarkable clinical significance for implementing preventive strategies and therapeutic targets in clinical settings.

## Results

### Baseline characteristics

A total of 8191 eligible subjects (3600 males, 4591 females) were included in the study with the average age of 61.74 ± 7.80 years. Baseline characteristics of the subjects based on direct bilirubin were presented in Table 1. Subjects with higher direct-bilirubin level at baseline were more likely to be with lower age, blood pressure, waist circumference, low density lipoprotein, triglyceride, total cholesterol, alanine aminotransferase, alanine aminotransferase, alkaline phosphatase, less smoking, less alcohol drinking, and more physical activity. Baseline characteristics based on indirect-bilirubin or total-bilirubin level was shown respectively in the Supplementary Table S3–S4. Moreover, the associations of NAFLD with baseline variables were presented in Supplementary Table S1. Subjects with newly developed NAFLD were more likely to be with higher level of blood pressure, waist circumference, low-density lipoprotein, triglyceride, total cholesterol, aspartate aminotransferase, alanine aminotransferase, alkaline phosphatase, lower level of direct bilirubin and high-density lipoprotein, and more often tend to occur smoking, alcohol drinking, diabetes, self-reported coronary heart disease, hypertension, and metabolic syndrome, compared with those which not developed NAFLD subjects. Additionally, significant difference were found between subjects who were included and excluded (Supplementary Table S2).

### Association between serum bilirubin levels and NAFLD risk

**Figure 1:**
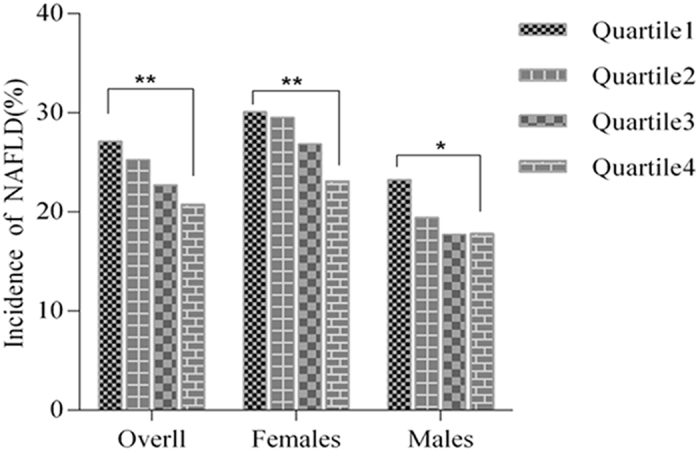
The incidence rates of NAFLD according to serum direct bilirubin levels quartiles. The quartiles of serum direct bilirubin levels were calculated by sex respectively and the cutoff values of serum direct bilirubin quartiles were <3.7, 3.7–4.5, 4.5–5.6, and >=5.6 umol/L for males and <2.9, 2.9–3.6, 3.6–4.5, and >=4.5 umol/L for females respectively. **p < 0.001 *p < 0.05.

The incidence of NAFLD according to quartiles of the serum direct bilirubin levels among study participants was shown in Fig. 1. In 2013, we observed 1956 cases of newly developed NAFLD with the overall incidence of 23.88% during the follow up of 5 years. Interestingly, an inverse association of direct bilirubin with incidence of NAFLD was presented, with significant dose-response relationship (*P* value < 0.05). When stratified by sex, the similarly significant trends were also found in both male and female subgroups (Fig. 1). However, the incidence of NAFLD by quartiles of the serum indirect or total bilirubin levels did not exhibit this trend (Supplementary Figure S1).

The associations of bilirubin (direct, indirect, total) with the risk of NAFLD were described in Table 2. In univariate regression model, the elevated bilirubin levels were significantly correlated with decreased NAFLD risk in a dose response manner. Compared with quartile 1 of direct bilirubin level, the ORs (95% CIs) for NAFLD among participants in quartile 3 and 4 quartile were 0.789 (0.685–0.910) and 0.704 (0.609–0.815), respectively, *P* value for trend < 0.001). Furthermore, after further adjustment for possible confounders, including social-demographic variables, anthropometric and biochemical parameters, and diseases history, the inverse associations were found to be slightly attenuated, but still significant and the dose response manner also consistently persisted. Correspondingly, subjects in the quartile 3 to 4 of direct bilirubin level had a significant decreased risk of NAFLD, approximately 21.2% and 31.2% respectively, compared with subjects in the lowest direct bilirubin quartile (adjusted ORs (95% CIs) for NAFLD were 0.843 (0.719–0.989), 0.768 (0.652–0.905) in quartile 3 and 4 compared with quartile 1 of direct bilirubin level, respectively, *P* value for trend 0.002). However, no significant associations were found between total and indirect bilirubin and the risk of NAFLD respectively.

### Stratified analysis according to sex and BMI

**Figure 2:**
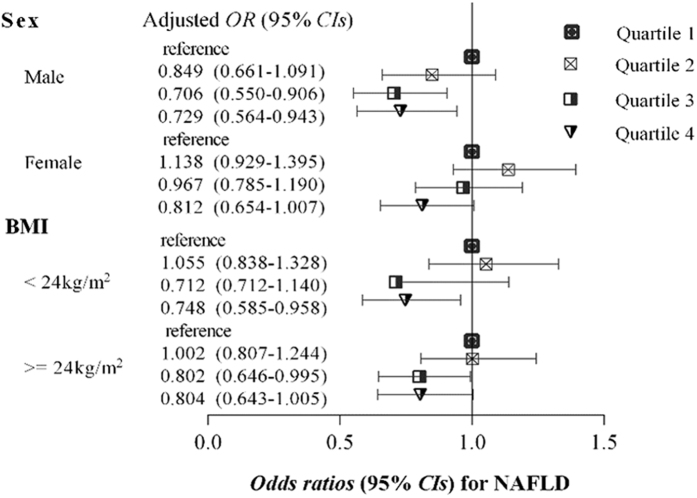
Multivariable-adjusted Odds ratios (95% *CIs*) for NAFLD based on serum direct bilirubin levels quartiles, stratified by sex and BMI respectively. The *ORs* (95% *CIs*) were presented compared with the quartile 1 of serum bilirubin level (reference), after adjustment for underlying confounders including age, education level, current smoking status, current alcohol drinking status, physical activity, coronary heart disease, diabetes, hypertension disease, tumor history, lipid lowering agent, waist circumference, body mass index, glucose, high-density lipoprotein, total cholesterol, triglyceride and uric acid.

In the sex-stratified analysis (Fig. 2 and Supplementary Table S5–S6), the inverse association and dose-response relationship between direct bilirubin levels and NAFLD risk were more significant in males compared with females. An approximately 26.1% reduced risk of NAFLD had been found in individuals with the highest direct bilirubin quartile compared with those with the lowest quartile of direct bilirubin (adjusted OR (95% CI) for NAFLD was 0.729 (0.564–0.943), *P* value for trend 0.015). In female individuals, the significant association was slightly attenuated after adjustment for possible confounders, but still presented a protective trend (adjusted OR (95% CI) for NAFLD was 0.812 (0.654–1.007) in the highest quartile compared with the lowest quartile of direct bilirubin, *P* value for trend 0.012). Additionally, no significant associations were presented between both total bilirubin and indirect bilirubin levels and the risk of NAFLD respectively (Supplementary Figure S2 and Supplementary Table S5–S6).

We also performed a stratified analysis according to BMI^27^,^28^ (Fig. 2 and Supplementary Table S7–S8). The correlation between elevated direct bilirubin and reduced NAFLD risk was more remarkable in individuals with BMI < 24 compared with those with BMI >=24. Correspondingly, subjects in the highest direct bilirubin quartile had an approximately 25.2% reduced risk of NAFLD compared with those in the lowest direct bilirubin quartile (adjusted OR (95% CI) for NAFLD was 0.748 (0.585–0.958), *P* value for trend 0.006). Among the individuals with BMI >=24, although the association was slightly attenuated, the elevated direct bilirubin levels still showed an inverse trend for NAFLD risk (adjusted OR (95% CI) for NAFLD was 0.804 (0.643–1.005) in the highest quartile versus the lowest quartile of direct bilirubin, *P* value for trend 0.022). With reference to the association of total or indirect bilirubin with NAFLD risk was not significant in BMI stratified analysis (Supplementary Figure S2 and Supplementary Table S7–S8).

## Discussion

In this prospective cohort study, we found that direct bilirubin levels were significantly associated with decreased NAFLD risk, presenting a protective biomarker for NAFLD. More importantly, this association was independent of classical risk factors including liver enzymes, diabetes, metabolic syndrome features, coronary artery disease and other classical metabolic risk factors. Similarly, apparent inverse effects of direct bilirubin on risk of NAFLD were also presented when stratified by sex and BMI, which consequently indicated that the significant association was independent of BMI. However, the associations between both total and indirect bilirubin and risk of NAFLD were not significant, which might be partly attributed to direct bilirubin that is more easily soluble in serum and exerts as active form prior to indirect bilirubin^29^.

These results were in agreement with previous studies demonstrating that bilirubin exhibited repressive effects on NAFLD development^24^,^25^,^26^,^30^. Chang *et al*.^30^ showed an inverse relationship between the direct bilirubin level and the incidence of NAFLD in a prospective study. However, only middle-aged male individuals were included. Besides, there have been accumulative studies revealing an inverse association between bilirubin levels and NAFLD^24^,^25^,^26^.

The biological mechanisms underlying the inverse association of direct bilirubin with NAFLD risk have not been completely clarified. NAFLD is a complex disease precisely modulated by numerous mechanisms including environmental, gut microbial and metabolic factors^31^. Besides, with the recent advances in genome-wide association studies^32^,^33^, genetic susceptibility is also proved to play important roles in NAFLD^34^,^35^,^36^. There has been accumulating evidence documenting that oxidative stress was thought to be a causal trigger in the progression from benign steatosis to more advanced forms of NAFLD and reactive oxygen species derived from fatty acid oxidation also be considered to perpetuate the liver damage of NAFLD^9^,^15^,^37^. Reportedly, bilirubin, the end product of haem catabolism, has been distinctly found to possess potential antagonizing oxidative stress properties by acting as antioxidant and cytoprotectant *in vitro* and *in vivo*^12^,^13^, which was partly validated by a previous population based study revealing the reduction of oxidative stress markers production in individuals with Gilbert’s syndrome was probably the result of antioxidant capacity of bilirubin^38^. Additionally, bilirubin was also frequently demonstrated to exhibit its protective roles against some diseases related to oxidative stress, including diabetes, metabolic syndrome, coronary artery disease and atherosclerosis^16^,^17^,^18^,^19^. Thus, it can be conceivably speculated that bilirubin could be linked to decreased risk of NAFLD, a hepatic manifestation of metabolic syndrome, probably induced by inhibiting oxidative stress.

Additionally, another possible mechanism linking bilirubin and decreased risk of NAFLD was presented by inhibiting insulin resistance, which has been well documented to be a well-established risk factor for NAFLD and a novel biomarker for liver damage in patients with NAFLD^14^,^20^,^39^. Moreover, insulin resistance was also considered to trigger NAFLD pathogenesis with oxidative stress interdependency^40^. Intriguingly, recent evidence has raised concern that elevated bilirubin exerted its protective effects against insulin resistance, and remarkably improved the insulin sensitivity by up-regulating adiponectin production and peroxisome proliferators-activated receptors (PPARγ) levels^41^,^42^,^43^. In line with population based studies, Biliverdin, the precursor of bilirubin, protecting against the deterioration of glucose tolerance also been found in the mice model. Taken together, these findings provided evidence supporting elevated bilirubin could contribute to protection against the risk of NAFLD probably through inhibiting insulin resistance and altering glucose metabolism.

Finally, accumulating evidence also existed that bilirubin could contribute to reduced risk of NAFLD through the suppression of inflammation milieu or complement activation, and lipids accumulation, which have been frequently documented to play an important role in triggering the pathogenesis of NAFLD^44^,^45^,^46^. Reportedly, anti-inflammatory effects of bilirubin played vital roles in reducing pro-inflammatory cytokines production, such as interleukin-6, interleukin-1, which had been found to contribute to hepatic steatosis in the murine model of NAFLD^47^. Additionally, there has been growing evidence suggesting bilirubin can markedly influence the lipogenesis, lipolysis of adipose tissue, and free fatty acid metabolism *in vitro* and *in vivo*^29^,^48^,^49^.

Interestingly, compared with the male individuals, the inverse association between direct-bilirubin and the risk of NAFLD risk was attenuated in the females after adjustment for confounders, which can be partly explained by the fact that the majority of female individuals (approximately 87.8%) in the present study were in the stage of menopause along with estrogen level decline, which have been proved to aggravate hepatic steatosis induced by increase of lipogenesis and triglyceride accumulation within the liver^50^. Besides, another underlying mechanism might be attributed to different effects of sex steroids on bilirubin metabolism and a relatively higher level of daily bilirubin production in males^51^. In addition, there were significant differences between subjects who were included and excluded, which were attributed to the population selection in the cohort study enrolling subject free of NAFLD or other hepatobiliary diseases and excluding those with NAFLD and other hepatobiliary diseases.

Certainly, several limitations should be acknowledged here. Firstly, the diagnosis of NAFLD was based on B-type ultrasonography, which has been thought to be a widely acceptable and cost-effective tool for screening NAFLD in large epidemiological and clinical practice, with reasonable accuracy and sensitivity for detecting fatty liver^52^. However, the gold standard for diagnosing NAFLD and differentiating NAFLD subtype still is liver biopsy. Moreover, population enrolled in the cohort study mainly were middle-aged and elderly Chinese people, resulting in the findings could not be fully extended to general populations and other ethnic groups.

In conclusion, in this prospective cohort study we demonstrated that direct bilirubin levels were inversely associated with the risk of NAFLD independent of possible confounders and supported its role as a protective biomarker for NAFLD among large middle aged and elderly Chinese population, probably based on the endogenous antioxidation of bilirubin. Certainly, further researches are warranted to validate these findings and elucidate the precise mechanisms underlying this association in experimental and large scale population based studies.

## Methods

### Study participants and design

**Figure 3:**
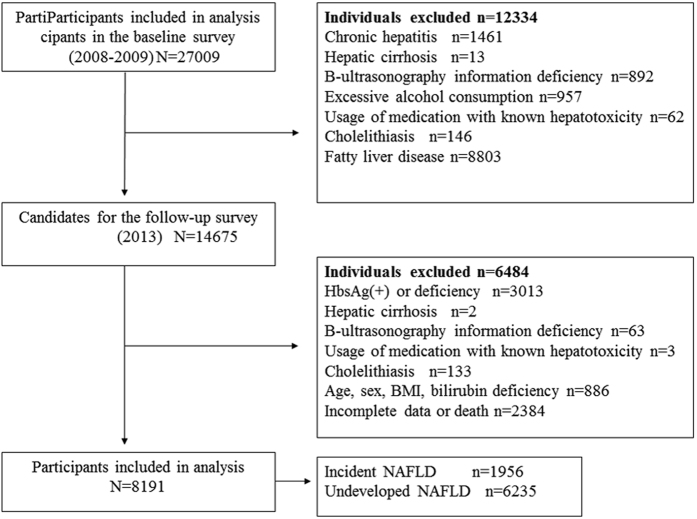
Flowchart of our prospective cohort study.

The study subjects and data were derived from the Dongfeng-Tongji (DFTJ) Cohort study, which was launched in 2008 recruiting retired employees of Dongfeng Motor Corporation (DMC) in Shiyan City, Hubei Province, The specific methods, design of study have been described previously^53^. In the initial stage of DFTJ cohort study, approximately 87% (n = 27009 out of 31000) of the recruited participants agreed to provide baseline blood samples and questionnaire information between 2008 and 2010. Subjects at baseline with presence of any of following conditions were excluded, including chronic hepatitis (n = 1461), hepatic cirrhosis (n = 13), abdominal B-type ultrasonography information deficiency (n = 892), excessive alcohol consumption (defined as more than 210 g/week for male or 140 g/week for female respectively^54^, n = 957), usage of medications with known hepatotoxicity within the past two weeks, such as valproate, amiodarone and tamoxifen (n = 62), cholelithiasis (n = 146). Moreover, 8803 subjects at baseline with fatty liver disease also were excluded. Among 14,675 participants who successfully finished the first follow-up in 2013, 6484 subjects were excluded with the presence of following diseases or factors, such as hepatitis B surface antigen positivity (HbsAg+) or information deficiency (n = 3013), hepatic cirrhosis (n = 2), abdominal B-type ultrasonography information deficiency (n = 63), usage of abovementioned medications (n = 3), cholelithiasis (n = 133), individuals without age, sex, BMI, and serum bilirubin information (n = 886). Additionally, 2384 subjects were removed from this study owing to death or lack of follow up data (n = 2384). Notably, it should be emphasized that some of these excluded individuals may simultaneously meet several exclusion criteria. Finally, the eligible sample size for analyses was 8189 in the present study (Fig. 3).

The cohort study has been approved by the Medical Ethics Committee of the School of Public Health, Tongji Medical College, Huazhong University of Science and Technology, Wuhan, China and Dongfeng General Hospital, DMC, Shiyan, China. Written informed consent was obtained from all participants and study protocols were carried out in accordance with the approved guidelines.

### Baseline measurement

Social-behavioral or sociodemographic information and medical history were collected through semi-structured questionnaires in the period of face to face interview, including age, sex, smoking status, alcohol consumption, education status, current medications status, physical activity, and history of diabetes, coronary heart disease, hypertension, tumor and digestive disease (gastrointestinal and hepatobiliary disease, chronic hepatitis disease). Anthropometric data was obtained by measuring waist circumference, standing height, weight, and blood pressure complying with standardized methods. Notably, during the process of measuring waist circumference, standing height and weight, the subjects were wore light clothes and barefoot. The measurement of blood pressure (BP) was measured in the participants’ right arm using a mercury sphygmomanometer. Notably, before the measurement of BP, the participants rested in the seated position for 10 minutes.

In addition, the hospital’ laboratory measured some biochemical markers according to the assay manual, such as, fasting plasma glucose was measured with Aeroset automatic analyzer (by glucose oxidase method; Abbott Laboratories. Abbott Park, Illinois, USA), low-density (LDL) lipoprotein, high-density lipoprotein (HDL), bilirubin (total, direct, indirect), total cholesterol, renal function (uric acid and blood urea nitrogen) and hepatic function (aspartate aminotransferase, alanine aminotransferase, alkaline phosphatase) was measured through ARCHITECT ci8200 automatic analyzer (Abbott, USA). Besides, the laboratory also provided the data of complete blood count, including haemoglobin, red blood cell count, and leukocyte count. The details can be available in a previous study^53^.

### Assessment of NAFLD, bilirubin, and covariates

NAFLD was identified as the presence of fatty liver disease (FLD) based on abdominal B-type ultrasound inspection after excluding excessive alcohol consumption (defined as more than 210 g/week for male or 140 g/week for female respectively^54^), and other conditions, such as hepatitis B infection (positive for hepatitis B surface antigen), medications with known hepatotoxicity (valproate, amiodarone and tamoxifen), chronic hepatitis, hepatic cirrhosis or carcinoma.

Serum bilirubin levels were categorized into four groups according to the quartiles of bilirubin levels, and were calculated by sex respectively. The cutoff values of bilirubin quartiles for males were direct bilirubin (<3.7, 3.7–4.5, 4.5–5.6, and >=5.6 umol/L), indirect bilirubin (<8.0, 8.0–10.6, 10.6–13.6, and >=13.6 umol/L), and total bilirubin (<12.0, 12.0–15.2, 15.2–19.0, and >=19.0 umol/L) respectively. The cutoff values of bilirubin levels for females were direct bilirubin (<2.9, 2.9–3.6, 3.6–4.5, and >=4.5 umol/L), indirect bilirubin (<6.6, 6.6–8.6, 8.6–11.2, and >=11.2 umol/L), and total bilirubin (<9.9, 9.9–12.0, 12.0–15.3, and >=15.3 umol/L) respectively.

With respect to assessment of the covariates, sociodemographic information was included in the questionnaire, such as sex, age, education level (defined as primary or middle/high/college or higher), drinking alcohol status (grouped as ex-drinker, current drinker, and nondrinker; current drinker defined as drinking at least one time per week for more than half of year), smoking status (classified as ex-smoker, current smoker, and nonsmoker; current smoker defined as smoking at least 1 cigarette per day for more than half of year), physical activity (dichotomized as yes or no, yes meaning those who exercise more than 20 min per day and more than three times per week in the previous six months). Some diseases were determined according to clinical diagnostic guideline and diseases history. Diabetes was determined as the presence of one of these conditions, including self-report diabetes history by physician’s diagnosis, usage of diabetes medication (insulin or glucose lowering agent) and fasting blood glucose level more than 7.0 mmol/L. The diagnosis of hypertension disease was based on occurring one of following conditions, including self-report hypertension history by physician’s diagnosis, current usage of antihypertensive medication, as well as systolic blood pressure >=140 mmHg or diastolic blood pressure >=90 mmHg for among both men and women. Coronary heart disease was determined by subjects’ self-reported. Metabolic syndrome was determined according to International Diabetes Foundation criteria of 2005^55^, including abdominal obesity (waist circumference ≥90 cm for men and ≥80 cm for women in Chinese) plus additional any 2 of the following 4 factors: (1) hypertriglyceridemia: fasting serum triglycerides ≥1.7 mmol/L; (2) high blood pressure: systolic ≥130 mm Hg, diastolic ≥85 mm Hg, or known treatment for hypertension; (3) hyperglycemia: fasting glucose level of ≥5.6 mmol/L (≥100 mg/dl) or known treatment for diabetes; and (4) low HDL cholesterol: fasting HDL cholesterol <1.0 mmol/L for male and <1.3 mmol/L for female; In addition, anthropometric data including waist circumference, weight, height, systolic and diastolic blood pressures, and biochemical parameters also were obtained by medical examination and laboratory inspection. Body mass index (BMI) was calculated as weight divided by height squared (kg/m^2^). Notably, these covariates’ definitions have been described clearly elsewhere^54^.

### Statistical analysis

Continuous variables were presented as mean ± SD, and categorical variables were expressed as percentages (%). The comparison of variables was performed using variance analysis for continuous variables and chi-square tests for categorical variables. We used univariate and multivariable logistic regression models to examine the association between bilirubin levels (direct, indirect, total) and NAFLD risk, and corresponding odds ratios (ORs) and 95% confidence intervals (CIs) were calculated simultaneously. Potential confounders influencing the risk of NAFLD that were adjusted in the multivariable models were social-demographic variables (age, sex, education levels, current smoking status, current alcohol drinking status, physical activity, lipid lowering agent), anthropometric and biochemical parameters (waist circumference, body mass index, glucose, high-density lipoprotein, total cholesterol, triglyceride and uric acid), and diseases history (coronary heart disease, diabetes, hypertension disease, tumor). All statistical analysis was carried out using SAS version 13.0 (SAS Institute, Cary, NC, USA), and a two-tailed *P* value of <0.05 was considered statistically significant.

## Additional Information

**How to cite this article**: Tian, J. *et al*. Association between bilirubin and risk of Non-Alcoholic Fatty Liver Disease based on a prospective cohort study. *Sci. Rep.* **6**, 31006; doi: 10.1038/srep31006 (2016).

## Supplementary Material

Supplementary Information

## Figures and Tables

**Table 1:** Baseline characteristics of study participants according to serum direct-bilirubin levels quartiles.

	Quartiles of serum direct-bilirubin levels (umol/L)				*P* value
	Q1	Q2	Q3	Q4	
Number	1967	2017	2125	2082	
Age^*^	61.32 (7.85)	61.79 (7.74)	62.27 (7.68)	61.57 (7.92)	<0.001^*a*^
Sex (female/male)%	56.63/43.37	57.61/42.39	54.31/45.69	55.76/44.24	0.175^*b*^
Waist (cm)^*^	80.08 (8.32)	79.35 (8.60)	80.37 (8.91)	80.39 (8.82)	<0.001^*a*^
BMI (kg/m^2^)^*^	23.34 (2.83)	23.24 (2.88)	23.37 (2.80)	23.22 (2.88)	0.268^*a*^
Blood pressure (mm Hg)^*^					
Systolic	126.15 (18.34)	126.00 (17.75)	127.40 (18.02)	127.64 (18.62)	0.004^*a*^
Diastolic	75.40 (10.50)	74.90 (10.36)	76.26 (10.62)	77.56 (10.92)	<0.001^*a*^
Fasting blood glucose (mmol/L)^*^	5.85 (1.47)	5.79 (1.23)	5.82 (1.37)	5.70 (1.48)	0.004^*a*^
Total bilirubin (umol/L)^*^	10.07 (2.90)	12.31 (2.65)	14.96 (3.54)	20.06 (7.32)	<0.001^*a*^
Direct bilirubin (umol/L)^*^	2.55 (0.72)	3.55 (0.47)	4.43 (0.56)	6.36 (1.81)	<0.001^*a*^
Indirect bilirubin (umol/L)^*^	7.52 (2.90)	8.76 (2.41)	10.54 (3.25)	13.71 (6.45)	<0.001^*a*^
HDL (mmol/L)^*^	1.38 (0.34)	1.47 (0.43)	1.49 (0.39)	1.49 (0.47)	<0.001^*a*^
LDL (mmol/L)^*^	3.19 (0.82)	3.05 (0.78)	2.99 (0.77)	2.84 (0.77)	<0.001^*a*^
Triglyceride (mmol/L)^*^	1.36 (1.00)	1.18 (0.60)	1.16 (0.59)	1.18 (0.72)	<0.001^*a*^
Total cholesterol (mmol/L)^*^	5.29 (0.97)	5.17 (0.92)	5.08 (0.92)	4.88 (0.93)	<0.001^*a*^
Uric acid (umol/L)^*^	286.40 (74.10)	280.74 (75.63)	285.35 (79.30)	276.16 (78.39)	<0.001^*a*^
AST (uL)^*^	22.76 (7.11)	23.68 (10.18)	23.52 (7.00)	25.19 (16.80)	<0.001^*a*^
ALT (uL)^*^	21.13 (12.65)	21.15 (14.41)	20.84 (9.99)	22.43 (25.02)	0.009^*a*^
ALP (uL)^*^	91.20 (25.95)	90.14 (29.93)	89.80 (24.83)	91.36 (36.13)	0.2348^*a*^
Hemoglobin (g/L)^*^	133.74 (14.22)	134.28 (13.18)	135.87 (13.45)	136.58 (14.58)	<0.001^*a*^
leukocyte (10^9^/L)^*^	5.93 (1.78)	5.88 (1.51)	5.88 (1.49)	5.89 (1.71)	0.747^*a*^
Education (primary or middle/high/college or-higher), %	64.88/24.52/10.59	62.23/26.01/11.76	64.95/23.29/11.76	68.36/21.96/9.68	0.004^*b*^
Physical activity (no/yes), %	18.86/81.14	15.91/84.09	14.73/85.27	15.99/84.01	0.004^*b*^
Smoking (current /ex-smoker/never), %	18.50/9.23/72.27	16.14/9.97/73.89/	17.07/12.12/70.82	15.89/12.12/71.99	0.005^*b*^
Alcohol drinking (current/ex-drinker/ never ), %	17.60/5.39/77.01	18.15/5.60/76.25	18.93/5.70/75.38	20.65/4.61/74.74	0.152^*b*^
Disease history(no/yes), %					
Diabetes mellitus, %	86.07/13.93	86.37/13.63	86.64/13.36	87.13/12.87	0.787^*b*^
Coronary heart disease, %	87.24/12.76	87.66/12.34	86.09/13.91	86.80/13.20	0.489^*b*^
Hypertension, %	55.52/44.48	55.97/44.03	53.18/46.82	53.12/46.88	0.130^*b*^
Tumor, %	95.78/4.22	94.25/5.75	95.80/4.20	96.44/3.56	0.006^*b*^
Metabolic syndrome(no/yes), %	81.36/18.64	83.81/16.19	82.74/17.26	81.84/18.16	0.183^*b*^
Medication history.(no/yes), %					
Lipid lowering agent	89.12/10.88	88.70/11.30	88.94/11.06	89.82/10.18	0.686^*b*^
Blood pressure lowering agent	76.21/23.79	75.41/24.59	73.22/26.78	74.64/25.36	0.152^*b*^
Diuretics	97.86/2.14	98.36/1.64	97.88/2.12	98.66/1.34	0.159^*b*^
Non-alcoholic fatty liver disease (no/yes), %	27.10/72.90	25.24/74.76	22.68/77.32	20.75/79.25	<0.001^b^

The quartiles of serum direct bilirubin levels were calculated by sex respectively and the cutoff values of serum direct bilirubin quartiles were <3.7, 3.7–4.5, 4.5–5.6, and >=5.6 umol/L for males and <2.9, 2.9–3.6, 3.6–4.5, and >=4.5 umol/L for females respectively.

Abbreviations: ALT, alanine aminotransferase; AST, aspartate aminotransferase; BMI, body mass index; ALP, alkaline phosphatase; HDL, high-density lipoprotein; LDL, low-density lipoprotein. ^*^Mean (standard deviation), ^a^Variance analysis for continuous data, ^b^Chi-square-tests for categorical data.

**Table 2:** Odds ratios (95% confidence intervals) for incident non-alcoholic fatty liver disease by serum bilirubin levels quartiles(n = 8191).

	Sample size(%)	Incident cases(%)	Univariate model	Age-and sex-adjusted	Multivariable model		
					Model 1^a^	Model 2^b^	Model 3^c^
Direct bilirubin (umol/L)							
Q1	1967 (24.01)	533 (27.10)	reference	reference	reference	reference	reference
Q2	2017 (24.62)	509 (25.24)	0.908 (0.788–1.046)	0.902 (0.783–1.040)	0.897 (0.778–1.035)	0.907 (0.784–1.048)	1.104 (0.867–1.187)
Q3	2125 (25.94)	482 (22.68)	0.789 (0.685–0.910)	0.794 (0.688–0.916)	0.785 (0.680–0.907)	0.788 (0.669–0.897)	0.843 (0.719–0.989)
Q4	2082 (25.42)	432 (20.75)	0.704 (0.609–0.815)	0.706 (0.610–0.815)	0.700 (0.604–0.811)	0.688 (0.593–0.799)	0.768 (0.652–0.905)
*P* for trend			<0.001	<0.001	<0.001	<0.001	0.002
Indirect bilirubin (umol/L)							
Q1	1987 (24.26)	500 (25.16)	reference	reference	reference	reference	reference
Q2	2092 (25.54)	459 (21.94)	0.836 (0.723–0.966)	0.840 (0.726–0.971)	0.841 (0.726–0.973)	0.839 (0.723–0.974)	0.822 (0.701–0.964)
Q3	2028 (24.76)	486 (23.96)	0.937 (0.812–1.082)	0.937 (0.811–1.082)	0.919 (0.794–1.063)	0.929 (0.801–1.077)	0.902 (0.769–1.057)
Q4	2084 (25.44)	511 (24.52)	0.966 (0.838–1.114)	0.969 (0.840–1.118)	0.970 (0.840–1.120)	0.990 (0.856–1.146)	0.962 (0.823–1.125)
*P* for trend			0.791	0.773	0.804	0.577	0.852
Total bilirubin (umol/L)							
Q1	2034 (24.83)	528 (25.96)	reference	reference	reference	reference	reference
Q2	1967 (24.01)	438 (5.35)	0.817 (0.707–0.945)	0.821 (0.710–0.950)	0.812 (0.701–0.941)	0.814 (0.701–0.772)	0.793 (0.676–0.931)
Q3	2110 (25.76)	511 (24.22)	0.912 (0.792–1.049)	0.905 (0.786–1.042)	0.897 (0.778–1.034)	0.892 (0.772–1.031)	0.901 (0.771–1.052)
Q4	2080 (25.39)	479 (23.03)	0.853 (0.740–0.984)	0.854 (0.740–0.985)	0.853 (0.739–0.985)	0.865 (0.747–1.001)	0.869 (0.743–1.017)
*P* for trend			0.137	0.131	0.144	0.202	0.328

The *ORs* and 95% *CIs* were calculated by unconditional logistic regression after adjusting for above potential confounders. The quartiles of serum bilirubin levels were calculated by sex respectively and the cutoff values of bilirubin quartiles for males were direct bilirubin (<3.7, 3.7–4.5, 4.5–5.6, and >=5.6 umol/L), indirect bilirubin (<8.0, 8.0–10.6, 10.6–13.6, and >=13.6 umol/L), and total bilirubin (<12.0, 12.0–15.2, 15.2–19.0, and >=19.0 umol/L) respectively, and for female were direct bilirubin (<2.9, 2.9–3.6, 3.6–4.5, and >=4.5 umol/L), indirect bilirubin (<6.6, 6.6–8.6, 8.6–11.2, and >=11.2 umol/L), and total bilirubin (<9.9, 9.9–12.0, 12.0–15.3, and >=15.3 umol/L) respectively.

^a^Model 1: Adjusted for the age, sex, plus education level, current smoking status, current alcohol drinking status and physical activity.

^b^Model 2: Adjusted for the variables in the model 1 plus coronary heart disease, diabetes, hypertension disease, tumor history and lipid lowering agent.

^c^Model 3: Furthered adjusted for the same set of variables in the model 2 plus waist circumference, body mass index, glucose, high-density lipoprotein, total cholesterol, triglyceride and uric acid.
