# Supplementary material for: Association between bilirubin and risk of Non-Alcoholic Fatty Liver Disease based on a prospective cohort study
Source: Sci Rep. 2016 Aug 3;6:31006. doi: 10.1038/srep31006 (PMC4975069; doi:10.1038/srep31006)
Supplement: Supplementary Information [file srep31006-s1.pdf]

Association between bilirubin and risk of Non-Alcoholic Fatty Liver Disease based on a prospective cohort study

Jianbo Tian[1], Rong Zhong[1], Cheng Liu[1], Yuhan Tang[2], Jing Gong[1], Jiang Chang[1], Jiao Lou[1], Juntao Ke[1], Jiaoyuan Li[1], Yi Zhang[1], Yang Yang[1], Ying Zhu[1], Yajie Gong[1], Yanyan Xu[2], Peiyi Liu[2], Xiao Yu[2], Lin Xiao[2], Min Du[2], Ling Yang[3], Jing Yuan[4], Youjie Wang[5], Weihong Chen[4], Sheng Wei[1], Yuan Liang[4], Xiaomin Zhang[4], Meian He[4], Tangchun Wu[4], Ping Yao[2*], Xiaoping Miao[1*]

[1] Department of Epidemiology and Biostatistics and the Ministry of Education (MOE) Key Lab of Environment and Health, School of Public Health, Tongji Medical College, Huazhong University of Science and Technology, 13 Hangkong Rd, Wuhan, Hubei, China.

[2] Department of Nutrition and Food Hygiene, Hubei Key Laboratory of Food Nutrition and Safety and the Ministry of Education (MOE) Key Lab of Environment and Health, School of Public Health, Tongji Medical College, Huazhong University of Science and Technology, 13 Hangkong Rd, Wuhan, 430030, Hubei, China.

[3] Division of Gastroenterology, Department of Internal Medicine, Union Hospital, Tongji Medical College, Huazhong University of Science and Technology, Wuhan, Hubei Province, China.

[4] Institute of Occupational Medicine and the Ministry of Education (MOE) Key Lab of Environment and Health, School of Public Health, Tongji Medical College, Huazhong University of Science and Technology, 13 Hangkong Rd, Wuhan, 430030, Hubei, China.

[5] Department of Maternal and Child Health, School of Public Health, Tongji Medical College, Huazhong University of Science and Technology, 13 Hangkong Rd, Wuhan, 430030, China.

[*]Corresponding authORs:
Xiaoping Miao, Department of Epidemiology and Biostatistics and the Ministry of Education (MOE) Key Lab of Environment and Health, School of Public Health, Tongji Medical College, Huazhong University of Science and Technology, 13 Hangkong Rd, Wuhan, Hubei, China. E-mail: miaoxp@mail.hust.edu.cn; tel: 86-27-836-50744; Fax: 86-27-836-50744; Ping Yao, Department of Nutrition and Food Hygiene, Hubei Key Laboratory of Food Nutrition and Safety and the Ministry of Education (MOE) Key Lab of Environment and Health, School of Public Health, Tongji Medical College, Huazhong University of Science and Technology, 13 Hangkong Rd, Wuhan, 430030, Hubei, China. E-mail:yaoping@mails.tjmu.cn; Tel: 0086-27-83650523; Fax: 0086-27-83650522.

Correspondence and requests for materials should be addressed to X.M. (miaoxp@mail.hust.edu.cn) or P.Y. (yaoping@mails.tjmu.cn).

Supplementary table S 1. Comparison of baseline characteristics of subjects developed and not developed NAFLD

	All participants	NAFLD		*P* value
		Developed	Not developed	
Number	8191	1956	6235	
Age*	61.74(7.80)	61.34(7.73)	61.87(7.82)	0.008[a]
Sex(female/male)%	56.03/43.95	64.21/35.79	53.49/46.51	<0.001[b]
Waist(cm)*	80.05(8.68)	83.45(8.21)	78.99(8.55)	<0.001[a]
BMI(kg/m^2)*	23.29(2.84)	24.87(2.71)	22.77(2.70)	<0.001[a]
Blood pressure(mm Hg)*				
Systolic	126.82(18.20)	128.28(18.06)	126.36(18.22)	<0.001[a]
Diastolic	76.05(10.65)	76.87(10.65)	75.80(10.64)	<0.001[a]
Fasting blood glucose(mmol/L)*	5.79(1.39)	5.91(1.35)	5.75(1.40)	<0.001[a]
Total bilirubin(umol/L)*	14.43(5.87)	14.04(6.15)	14.55.(5.77)	<0.001[a]
Direct bilirubin(umol/L)*	4.25(1.75)	4.01(1.61)	4.33(1.78)	<0.001[a]
Indirect bilirubin(umol/L)*	10.18(4.71)	10.03(4.96)	10.23(4.62)	0.122[a]
HDL(mmol/L)*	1.46(0.41)	1.40(0.36)	1.47(0.43)	<0.001[a]
LDL(mmol/L)*	3.01(0.80)	3.07(0.83)	3.00(0.79)	<0.001[a]
Triglyceride(mmol/L)*	1.22(0.74)	1.43(0.97)	1.15(0.65)	<0.001[a]
Total cholesterol(mmol/L)*	5.10(0.94)	5.19(0.96)	5.07(0.94)	<0.001[a]
Uric acid(umol/L)*	282.13(77.04)	291.90(77.12)	279.06(76.76)	<0.001[a]
AST(u/L)*	23.80(11.08)	23.18(8.09)	24.00(11.38)	<0.001[a]
ALT(u/L)*	21.39(16.58)	22.59(13.81)	21.02(17.34)	<0.001[a]
ALP(u/L)*	90.61(29.57)	91.75(26.93)	90.26(30.34）	0.039[a]
Hemoglobin(g/L)*	135.15(13.91)	135.23(13.84)	135.13(13.93)	0.787[a]
leukocyte(10^9 L)*	5.89(1.62)	6.04(1.75)	5.85(1.58)	<0.001[a]
Education (primary or middle/high/college or-higher),%	65.13/23.92/10.95	67.37/22.34/10.29	64.43/24.42/11.16	0.060[b]
Physical activity (no/yes),%	16.34/83.66	17.59/82.41	15.94/84.06	0.086[b]
Smoking (current/ex-smoker/never),%	16.89/10.89/72.22	14.30/9.38/76.32	17.70/11.37/70.93	<0.001[b]
Alcohol drinking (current/ex-drinker/never),%	18.85/5.32/75.82	17.54/4.60/77.85	19.27/5.55/75.18	0.044[b]
Disease history(no/yes),%				
Diabetes mellitus	86.56/13.44	83.28/16.72	87.59/12.41	<0.001[b]
Coronary heart disease	86.93/13.07	84.29/15.71	87.76/12.24	<0.001[b]
Hypertension	54.41/45.59	45.09/54.91	57.34/42.66	<0.001[b]
Tumor	95.57/4.43	96.06/3.94	95.42/4.58	0.231[b]
Metabolic syndrome	82.44/17.56	69.58/30.42	86.48/13.52	<0.001[b]
Medication history ,(no/yes),%				
Lipid lowering agent	74.84/25.16	67.59/32.41	77.11/22.89	<0.001[b]
Blood pressure lowering agent	89.15/10.85	85.79/14.21	90.20/9.80	<0.001[b]
Diuretics	98.19/1.81	97.60/2.40	98.38/1.62	0.233[b]

* Mean (standard deviation), [a] Variance analysis for continuous data, [b] Chi-square-tests for categorical data. Abbreviations: ALT, alanine aminotransferase; AST, aspartate aminotransferase; BMI, body mass index; ALP, alkaline phosphatase; HDL, high-density lipoprotein; LDL, low-density lipoprotein.

Supplementary table S 2. Comparison of baseline characteristics of subjects included and excluded.

	Included	Excluded	*P* value
Number	8191	18818	
Age*	61.74(7.80)	62.88(7.78)	<0.001[a]
Sex(female/male)	56.03/43.95	55.10/44.90	0.148[b]
Waist(cm)*	80.05(8.68)	84.68(9.54)	<0.001[a]
BMI(kg/m2)*	23.29(2.84)	25.32(3.45)	<0.001[a]
Blood pressure(mm Hg)*			
Systolic	126.82(18.20)	131.33(18.83)	<0.001[a]
Diastolic	76.05(10.65)	78.73(10.88)	<0.001[a]
Fasting blood glucose(mmol/L)*	5.79(1.39)	6.33(2.19)	<0.001[a]
Total bilirubin(umol/L)*	14.43(5.87)	14.18.(5.83)	0.035[a]
Direct bilirubin(umol/L)*	4.25(1.75)	3.94(2.26)	<0.001[a]
Indirect bilirubin(umol/L)*	10.18(4.71)	10.24(4.77)	<0.001[a]
HDL(mmol/L)*	1.46(0.41)	1.43(0.41)	<0.001[a]
LDL(mmol/L)*	3.01(0.80)	3.02(0.86)	0.540[a]
Triglyceride(mmol/L)*	1.22(0.74)	1.58(1.28)	<0.001[a]
Total cholesterol(mmol/L)*	5.10(0.94)	5.21(0.10)	<0.001[a]
Uric acid(umol/L)*	282.13(77.04)	306.46(86.22)	<0.001[a]
AST(uL)*	23.80(11.08)	26.26(16.77)	<0.001[a]
ALT(uL)*	21.39(16.58)	25.83(21.38)	<0.001[a]
ALP(uL)*	90.61(29.57)	91.54(37.19）	0.052[a]
Hemoglobin(g/L)*	135.15(13.91)	137.43(14.75)	<0.001[a]
leukocyte(*10^9L)*	5.89(1.62)	6.17(1.70)	<0.001[a]
Education (primary or middle/high/college or-higher),%	65.13/23.92/10.95	65.23/24.30/10.48	0.700[b]
Smoking (current/ex-smoker/never),%	16.89/10.89/72.22	17.70/11.37/70.93	0.698[b]
Alcohol drinking (current/ex-drinker/never),%	18.85/5.32/75.82	20.01/5.51/74.48	0.061[b]

* Mean (standard deviation), [a] Variance analysis for continuous data, [b] Chi-square-tests for categorical data.

Abbreviations: ALT, alanine aminotransferase; AST, aspartate aminotransferase; BMI, body mass index; ALP, alkaline phosphatase; HDL, high-density lipoprotein; LDL, low-density lipoprotein.

Supplementary table S 3. Baseline characteristics of study participants according to serum indirect-bilirubin levels quartiles

	Quartiles of serum indirect-bilirubin (umol/L)				*P* value
	Q1	Q2	Q3	Q4	
Number	1987	2092	2028	2084	
Age*	61.33(7.84)	62.12(7.92)	61.96(7.68)	61.55(7.750	0.004[a]
Sex(female/male)%	56.29/43.71		57.44/42.56	8011.00	0.301[b]
Waist(cm)*	79.73(8.16)	79.97(8.97)	80.04(8.89)	80.11(8.66)	0.101[a]
BMI(kg/m^2)*	23.21(2.80)	23.30(2.88)	23.35(2.86)	23.31(2.84）	0.478[a]
Blood pressure(mm Hg)*					
Systolic	126.67(18.62)	127.00(17.61)	127.31(18.51)	126.28(18.06)	0.304[a]
Diastolic	75.91(10.68)	76.18(10.53)	76.14(10.90)	75.98(10.51)	0.833[a]
Fasting blood glucose(mmol/L)*	5.78(1.43)	5.72(1.13)	5.80(1.36)	5.86(1.60)	0.022[a]
Total bilirubin(umol/L)*	9.00(1.88)	12.07(1.84)	15.00(2.34)	21.42(6.34)	<0.001[a]
Direct bilirubin(umol/L)*	3.49(1.53)	3.77(1.17)	4.28(1.42)	5.43(2.06)	<0.001[a]
Indirect bilirubin(umol/L)*	5.53(1.31)	8.29(1.09)	10.72(1.37)	15.99(4.91)	<0.001[a]
HDL(mmol/L)*	1.45(0.42)	1.46(0.46)	1.45(0.38)	1.46(0.38)	0.905[a]
LDL(mmol/L)*	2.97(0.80)	3.01(0.79)	3.03(0.78)	3.04(0.81)	0.027[a]
Triglyceride(mmol/L)*	1.25(0.79)	1.21(0.83)	1.20(0.64)	1.22(0.69)	0.108[a]
Total cholesterol(mmol/L)*	5.05(0.96)	5.11(0.94)	5.11(0.92)	5.13(0.95)	0.064[a]
Uric acid(umol/L)*	279.05(78.09)	282.08(76.73)	282.14(76.26)	285.11(77.03)	0.099[a]
AST(u/L)*	23.55(8.80)	23.66(9.53)	23.45(8.20)	24.52(15.87)	0.007[a]
ALT(u/L)*	21.22(12.49)	21.18(13.51)	20.87(12.71)	22.27(24.30)	0.038[a]
ALP(u/L)*	92.59(30.26)	90.29(32.90)	89.98(28.54)	89.68(26.07)	0.007[a]
Hemoglobin(g/L)*	131.65(14.01)	134.78(13.56)	136.32(13.46)	137.77(13.88)	<0.001[a]
leukocyte(10^9 L)*	6.10(1.55)	5.87(1.51)	5.86(1.74)	5.75(1.68)	<0.001[a]
Education (primary or middle/high/college or-higher),%	65.89/23.74/10.37	64.71/24.06/11.23	65.06/23.64/11.30	64.91/24.21/10.87	0.965[b]
Physical activity (no/yes),%	18.02/81.98	16.83/83.17	15.78/84.22	14.78/85.22	0.035[b]
Smoking(current/ex-smoker/never),%	20.80/9.94/69.26	17.69/9.76/72.55	14.54/11.23/74.23	14.63/12.61/72.76	<0.001[b]
Alcohol drinking (current/ex-drinker/never),%	18.22/5.74/76.04	17.93/5.36/76.71	19.49/5.48/75.04	19.77/4.75/75.48	0.523[b]

Disease history(no/yes),%					
Diabetes mellitus,%	85.51/14.49	87.86/12.14	86.44/13.56	86.37/13.63	0.171[b]
Coronary heart disease,%	87.40/12.60	87.15/12.85	85.75/14.25	87.43/12.57	0.339[b]
Hypertension,%	54.35/45.65	13.75/11.79	53.75/46.25	55.71/44.29	0.558[b]
Tumor,%	95.43/4.57	95.55/4.45	94.85/5.15	96.44/3.56	0.104[b]
Metabolic syndrome(no/yes),%	82.40/17.60	83.14/16.86	81.84/18.16	82.38/17.62	0.749[b]
Medication history ,(no/yes),%					
Lipid lowering agent	88.27/11.73	89.63/10.37	88.71/11.29	89.92/10.08	0.288[b]
Blood pressure lowering agent	75.54/24.46	74.33/25.67	74.31/25.69	75.19/24.81	0.742[b]
Diuretics	98.09/1.91	97.75/2.25	98.27/1.73	98.66/1.34	0.172[b]
Non-alcoholic fatty liver disease(no/yes),%	74.84/25.16	78.06/21.94	76.04/23.96	75.48/24.52	0.086[b]

* Mean (standard deviation), [a] Variance analysis for continuous data, [b] Chi-square-tests for categorical data. The quartiles of serum indirect bilirubin levels were calculated by sex respectively. The cutoff values of serum indirect bilirubin quartiles were <8.0, 8.0–10.6, 10.6–13.6, and >=13.6 umol/L for males and <6.6, 6.6–8.6, 8.6–11.2, and >=11.2 umol/L for females respectively.

Abbreviations: ALT, alanine aminotransferase; AST, aspartate aminotransferase; BMI, body mass index; ALP, alkaline phosphatase; HDL, high-density lipoprotein; LDL, low-density lipoprotein.

Supplementary table S 4. Baseline characteristics of study participants according to serum total-bilirubin levels quartiles

	Quartiles of serum total-bilirubin (umol/L)				*P* value
	Q1	Q2	Q3	Q4	
Number	2034	1907	2110	2080	
Age*	61.46(7.88)	62.06(7.82)	61.91(7.80)	61.54(7.71)	0.041[a]
Sex(female/male)%	56.29/43.71	54.50/45.50	57.44/42.56	55.87/44.13	0.301[b]
Waist(cm)*	79.69(8.22)	80.30(8.97)	80.04(8.77)	80.19(8.75)	0.138[a]
BMI(kg/m^2)*	23.34(2.83)	23.36(2.86)	23.31(2.85)	23.29(2.83)	0.472[a]
Blood pressure(mm Hg)*					
Systolic	126.49(18.35)	126.93(17.87)	127.32(18.36)	126.52(18.19)	0.480[a]
Diastolic	75.50(10.59)	76.21(10.50)	76.25(10.70)	76.24(10.78)	0.067[a]
Fasting blood glucose(mmol/L)*	5.82(1.39)	5.74(1.26)	5.76(1.29)	5.84(1.60)	0.070[a]
Total bilirubin(umol/L)*	8.83(1.65)	12.07(1.53)	14.90(2.01)	21.66(6.18)	<0.001[a]
Direct bilirubin(umol/L)*	3.05(1.24)	3.80(1.09)	4.28(1.21)	5.82(1.96)	<0.001[a]
Indirect bilirubin(umol/L)*	5.80(1.49)	8.27(1.47)	10.62(1.76)	15.83(5.05)	<0.001[a]
HDL(mmol/L)*	1.43(0.38)	1.46(0.47)	1.46(0.39)	1.47(0.40)	0.025[a]
LDL(mmol/L)*	3.03(0.81)	3.02(0.79)	3.04(0.79)	2.97(0.80)	0.038[a]
Triglyceride(mmol/L)*	1.26(0.93)	1.22(0.68)	1.21(0.66)	1.19(067)	0.012[a]
Total cholesterol(mmol/L)*	5.12(0.98)	5.10(0.92)	5.13(0.94)	5.05(0.95)	0.028[a]
Uric acid(umol/L)*	282.51(76.24)	282.15(78.29)	282.29(76.27)	281.58(77.44)	0.983[a]
AST(u/L)*	23.14(8.17)	23.87(9.27)	23.53(8.83)	24.67(16.03)	<0.001[a]
ALT(u/L)*	21.00(12.11)	21.50(15.21)	20.76(10.52)	22.32(24.63)	0.013[a]
ALP(u/L)*	92.08(29.65)	90.40(26.05)	89.87(33.12）	90.13(28.75)	0.072[a]
Hemoglobin(g/L)*	131.71(14.04)	135.16(13.45)	136.20(13.27)	137.47(14.19)	<0.001[a]
leukocyte(10^9 L)*	6.03(1.56)	5.94(1.73)	5.83(1.50)	5.78(1.69)	<0.001[a]
Education (primary or middle/high/college or-higher), %	64.68/24.55/10.76	65.49/23.33/11.18	64.88/23.72/11.40	16.66/6.12/2.66	0.933[b]
Physical activity (no/yes),%	18.86/81.14	15.91/84.09	14.73/85.27	15.99/84.01	0.004[b]
Smoking(current/ex-smoker/never),%	20.77/9.42/69.81	17.78/10.68/71.54	14.49/11.26/74.25	14.67/12.16/73.17	<0.001[b]
Alcohol drinking (current/ex-drinker/never),%	17.51/5.66/76.83	19.37/5.54/75.09	19.01/5.31/75.68	19.52/4.81/75.67	0.569[b]
Disease history(no/yes),%					

Diabetes mellitus,%	85.20/14.80	21.15/2.87	86.78/13.22	86.25/13.75	0.064[b]
Coronary heart disease,%	87.44/12.56	87.20/12.80	85.79/14.21	85.79/14.21	0.350[b]
Hypertension,%	54.47/15.53	55.52/44.48	51.75/48.25	56.01/43.99	0.028[b]
Tumor,%	95.23/4.77	95.16/4.84	95.58/4.42	96.28/3.72	0.292[b]
Metabolic syndrome(no/yes),%	82.25/17.75	82.69/17.31	82.27/17.73	82.58/17.42	0.977[b]
Medication history ,(no/yes),%					
Lipid lowering agent	88.45/11.55	89.48/10.52	88.77/11.23	89.90/10.10	0.424[b]
Blood pressure lowering agent	75.27/24.73	74.73/25.27	73.74/26.26	75.63/24.38	0.524[b]
Diuretics	97.84/2.16	98.12/1.88	98.25/1.75	98.56/1.44	0.375[b]
Non-alcoholic fatty liver disease(no/yes),%	74.04/25.96	77.73/22.27	75.78/24.22	76.97/23.03	0.035[b]

* Mean (standard deviation), [a] Variance analysis for continuous data, [b] Chi-square-tests for categorical data. The quartiles of serum total bilirubin levels were calculated by sex respectively. The cutoff values of serum total bilirubin quartiles were <12.0, 12.0–15.2, 115.2–19.0, and >=19.0 umol/L in males and<9.9, 9.9–12.0, 12.0–15.3, and >=15.3 umol/L in females respectively.

Abbreviations: ALT, alanine aminotransferase; AST, aspartate aminotransferase; BMI, body mass index; ALP, alkaline phosphatase; HDL, high-density lipoprotein; LDL, low-density lipoprotein

Supplementary table S 5. Odds ratios (95% confidence intervals) for incident NAFLD according to serum bilirubin levels quartiles in males (n=3600)

	Sample size (%)	Incident cases (%)	Univariate model	Age-and sex-adjusted	Multivariable model		
					Model 1 [a]	Model 2 [b]	Model 3 [c]
Direct bilirubin(umol/L)							
Q1	853(23.69)	198(23.21)	reference	reference	reference	reference	reference
Q2	855(23.75)	166(19.42)	0.797(0.632-1.005)	0.795(0.630-1.003)	0.801(0.634-1.012)	0.804(0.635-1.017)	0.849(0.661-1.091)
Q3	971(26.97)	172(17.71)	0.712(0.566-0.895)	0.711(0.565-0.894)	0.701(0.556-0.883)	0.712(0.564-0.899)	0.706(0.550-0.906)
Q4	921(25.58)	164(17.81)	0.717(0.568-0.904)	0.716(0.568-0.903)	0.717(0.568-0.906)	0.727(0.574-0.920)	0.729 (0.564-0.943)
P for trend			0.005	0.005	0.008	0.012	0.015
Indirect bilirubin(umol/L)							
Q1	858(23.83)	175(20.40)	reference	reference	reference	reference	reference
Q2	940(26.11)	179(19.04)	0.918(0.728-1.158)	0.917(0.726-1.157)	0.919(0.727-1.162)	0.912(0.720-1.156)	0.849(0.661-1.089)
Q3	883(24.53)	165(18.69)	0.897(0.708-1.137)	0.896(0.706-1.135)	0.877(0.690-1.115)	0.880(0.691-1.120)	0.828(0.642-1.069)
Q4	919(25.53)	181(19.70)	0.957(0.759-1.208)	0.956(0.758-1.206)	0.955(0.755-1.207)	0.968(0.764-1.226)	0.855(0.666-1.096)
P for trend			0.795	0.791	0.758	0.867	0.299
Total bilirubin(umol/L)							
Q1	889(24.69)	189(21.26)	reference	reference	reference	reference	reference
Q2	895(24.86)	168(18.77)	0.856(0.678-1.080)	0.855(0.677-1.078)	0.845(0.668-1.068)	0.861(0.679-1.091)	0.774(0.603-0.994)
Q3	898(24.94)	170(18.93)	0.865(0.686-1.090)	0.864(0.685-1.089)	0.855(0.676-1.080)	0.871(0.688-1.102)	0.839(0.654-1.076)
Q4	918(25.50)	173(18.85)	0.860(0.683-1.083)	0.859(0.682-1.082)	0.855(0.678-1.079)	0.879(0.695-1.111)	0.790(0.616-1.012)
P for trend			0.286	0.285	0.281	0.416	0.165

[a] model 1：Adjusted for the age, plus education level, current smoking status, current alcohol drinking status and physical activity.

[b] model 2：Adjusted for the variables in the model 1 plus coronary heart disease, diabetes, hypertension disease, tumor history and lipid lowering agent.

[c] model 3：Furthered adjusted for the same set of variables in the model 2 plus waist circumference, body mass index, glucose, high-density lipoprotein, total cholesterol, triglyceride and uric acid.

The *ORs* and 95% *CIs* were calculated by unconditional logistic regression after adjusting for above potential confounders and the cutoff values of bilirubin quartiles for males were direct bilirubin (<3.7, 3.7–4.5, 4.5–5.6, and >=5.6 umol/L), indirect bilirubin (<8.0, 8.0–10.6, 10.6–13.6, and >=13.6 umol/L), and total bilirubin (<12.0, 12.0–15.2, 15.2–19.0, and >=19.0 umol/L) respectively.

Supplementary table S 6. Odds ratios (95% confidence intervals) for incident NAFLD according to serum bilirubin levels quartiles in females (n=4591)

	Sample size (%)	Incident cases (%)	Univariate model	Age-and sex-adjusted	Multivariable model		
					Model 1 [a]	Model 2 [b]	Model 3 [c]
Direct bilirubin(umol/L)							
Q1	1114(24.26)	335(30.07)	reference	reference	reference	reference	reference
Q2	1162(25.31)	343(29.52)	0.974(0.814-1.166)	0.973(0.813-1.165)	0.966(0.806-1.158)	0.981(0.815-1.180)	1.138(0.929-1.395)
Q3	1154(26.97)	172(17.71)	0.854(0.712-1.025)	0.852(0.710-1.023)	0.846(0.703-1.017)	0.822(0.0.729681-0.993)	0.967(0.785-1.190)
Q4	1161(25.29)	268(23.08)	0.698(0.579-0.842)	0.698(0.579-0.841)	0.685(0.567-0.827)	0.663(0.547-0.805)	0.812(0.654-1.007)
P for trend			<0.001	<0.001	<0.001	<0.001	0.012
Indirect bilirubin(umol/L)							
Q1	1129(24.59)	325(28.79)	reference	reference	reference	reference	reference
Q2	1152(25.09)	280(24.31)	0.794(0.659-0.957)	0.793(0.658-0.955)	0.795(0.659-0.959)	0.796(0.657-0.965)	0.811(0.658-0.999)
Q3	1145(24.94)	321(28.03)	0.964(0.803-1.156)	0.962(0.802-1.155)	0.947(0.788-1.139)	0.963(0.798-1.162)	0.953(0.777-1.170)
Q4	1165(25.38)	330(28.33)	0.978(0.816-1.172)	0.978(0.816-1.172)	0.983(0.819-1.179)	1.009(0.838-1.216)	1.062(0.867-1.301)
P for trend			0.578	0.574	0.565	0.374	0.218
Total bilirubin(umol/L)							
Q1	1145(24.94)	339(29.61)	reference	reference	reference	reference	reference
Q2	1072(23.35)	270(25.19)	0.800(0.664-0.965)	0.800(0.663-0.965)	0.795(0.658-0.960)	0.787(0.649-0.955)	0.814(0.660-1.004)
Q3	1212(26.40)	341(28.14)	0.931(0.779-1.112)	0.930(0.778-1.111)	0.925(0.772-1.107)	0.905(0.753-1.088)	0.944(0.773-1.154)
Q4	1162(25.31)	306(26.33)	0.850(0.708-1.020)	0.850(0.709-1.020)	0.853(0.710-1.025)	0.860(0.713-1.037)	0.952(0.776-1.167)
P for trend			0.239	0.241	0.267	0.351	0.903

[a] model 1：Adjusted for the age, plus education level, current smoking status, current alcohol drinking status and physical activity.

[b] model 2：Adjusted for the variables in the model 1 plus coronary heart disease, diabetes, hypertension disease, tumor history and lipid lowering agent .

[c] model 3：Furthered adjusted for the same set of variables in the model 2 plus waist circumference, body mass index, glucose, high-density lipoprotein, total cholesterol, triglyceride and uric acid.

The *ORs* and 95% *CIs* were calculated by unconditional logistic regression after adjusting for above potential confounders and the cutoff values of bilirubin quartiles for females were direct bilirubin (<2.9, 2.9–3.6, 3.6–4.5, and >=4.5 umol/L), indirect bilirubin (<6.6, 6.6–8.6, 8.6–11.2, and >=11.2 umol/L), and total bilirubin (<9.9, 9.9–12.0, 12.0–15.3, and >=15.3 umol/L) respectively.

Supplementary table S 7. Odds ratios (95% confidence intervals) for incident NAFLD by serum bilirubin levels quartiles among individuals with BMI<24 kg/m^2 (n=4973)

	Sample size (%)	Incident cases (%)	Univariate model	Age-and sex-adjusted	Multivariable model		
					Model 1 [a]	Model 2 [b]	Model 3 [c]
Direct bilirubin(umol/L)							
Q1	1176(23.65)	199(16.92)	reference	reference	reference	reference	reference
Q2	1248(25.38)	199(15.95)	0.931(0.751-1.155)	0.926(0.746-1.149)	0.908(0.731-1.128)	0.927(0.744-1.155)	1.055(0.838-1.328)
Q3	1268(25.50)	184(14.51)	0.833(0.670-1.037)	0.841(0.675-1.047)	0.827(0.663-1.031)	0.817(0.653-1.023)	0.712(0.712-1.140)
Q4	1281(25.76)	152(11.87)	0.661(0.526-0.830)	0.657(0.523-0.826)	0.659(0.524-0.829)	0.650(0.515-0.821)	0.748(0.585-0.958)
P for trend			0.0002	<0.001	<0.001	<0.001	0.006
Indirect bilirubin(umol/L)							
Q1	1219(24.51)	201(16.49)	reference	reference	reference	reference	reference
Q2	1272(25.58)	158(12.42)	0.718(0.574-0.900)	0.711(0.567-0.891)	0.708(0.564-0.888)	0.693(0.549-0.873)	0.706(0.556-0.896)
Q3	1213(24.39)	181(14.92)	0.888(0.714-1.105)	0.888(0.713-1.105)	0.864(0.692-1.079)	0.871(0.695-1.090)	0.883(0.700-1.113)
Q4	1027(25.48)	194(15.29)	0.914(0.737-1.133)	0.902(0.727-1.119)	0.906(0.729-1.125)	0.918(0.737-1.144)	0.882(0.702-1.109)
P for trend			0.970	0.959	0.971	0.897	0.785
Total bilirubin(umol/L)							
Q1	1255(23.43)	203(16.18)	reference	reference	reference	reference	reference
Q2	1165(25.84)	158(13.56)	0.813(0.649-1.018)	0.809(0.646-1.014)	0.798(0.635-1.002)	0.794(0.630-1.002)	0.819(0.645-1.040)
Q3	1285(15.49)	199(15.49)	0.950(0.767-1.175)	0.939(0.759-1.163)	0.931(0.750-1.155)	0.925(0.743-1.152)	0.952(0.759-1.194)
Q4	1268(25.50)	174(13.72)	0.824(0.662-1.027)	0.814(0.653-1.015)	0.822(0.658-1.025)	0.832(0.665-1.042)	0.841(0.666-1.061)
P for trend			0.214	0.176	0.219	0.268	0.297

[a] model 1：Adjusted for the age, plus education level, current smoking status, current alcohol drinking status and physical activity.

[b] model 2：Adjusted for the variables in the model 1 plus coronary heart disease, diabetes, hypertension disease, tumor history and lipid lowering agent.

[c] model 3：Furthered adjusted for the same set of variables in the model 2 plus waist circumference, body mass index, glucose, high-density lipoprotein, total -cholesterol, triglyceride and uric acid.

The *ORs* and 95% *CIs* were calculated by unconditional logistic regression after adjusting for above potential confounders. The quartiles of bilirubin levels were calculated by sex respectively. The cutoff values of bilirubin quartiles for males were direct bilirubin (<3.7, 3.7–4.5, 4.5–5.6, and >=5.6 umol/L), indirect bilirubin (<8.0, 8.0–10.6, 10.6–13.6, and >=13.6 umol/L), and total bilirubin (<12.0, 12.0–15.2, 15.2–19.0, and >=19.0 umol/L) respectively, and for females were direct bilirubin (<2.9, 2.9–3.6, 3.6–4.5, and >=4.5 umol/L), indirect bilirubin (<6.6, 6.6–8.6, 8.6–11.2, and >=11.2 umol/L), and total bilirubin (<9.9, 9.9–12.0, 12.0–15.3, and >=15.3 umol/L) respectively.

Supplementary table S 8. Odds ratios (95% confidence intervals) for incident NAFLD by serum bilirubin levels quartiles among individuals with BMI >=24 kg/m^2 (n=3218)

	Sample size (%)	Incident cases (%)	Univariate model	Age-and sex-adjusted	Multivariable model		
					Model 1 [a]	Model 2 [b]	Model 3 [c]
Direct bilirubin(umol/L)							
Q1	791(24.58)	334(42.23)	reference	reference	reference	reference	reference
Q2	769(23.90)	310(40.31)	0.924(0.755-1.131)	0.929(0.757-1.140)	0.945(0.768-1.162)	0.948(0.769-1.168)	1.002(0.807-1.244)
Q3	857(26.63)	298(34.77)	0.729(0.598-0.890)	0.743(0.607-0.910)	0.740(0.603-0.907)	0.743(0.604-0.914)	0.802(0.646-0.995)
Q4	801(24.89)	280(24.89)	0.735(0.601-0.900)	0.757(0.616-0.931)	0.763(0.620-0.939)	0.755(0.612-0.932)	0.804(0.643-1.005)
P for trend			0.001	0.003	0.003	0.003	0.022
Indirect bilirubin(umol/L)							
Q1	768(23.87)	299(38.93)	reference	reference	reference	reference	reference
Q2	820(25.48)	301(36.71)	0.910(0.743-1.114)	0.966(0.785-1.187)	0.985(0.800-1.213)	0.997(0.808-1.230)	0.971(0.782-1.205)
Q3	815(25.33)	305(37.42)	0.938(0.766-1.149)	0.950(0.773-1.168)	0.955(0.776-1.177)	0.978(0.793-1.207)	0.943(0.759-1.172)
Q4	815(25.33)	317(38.90)	0.998(0.816-1.222)	1.056(0.860-1.297)	1.078(0.876-1.326)	1.110(0.901-1.369)	1.074(0.865-1.333)
P for trend			0.816	0.549	0.462	0.313	0.488
Total bilirubin(umol/L)							
Q1	779(24.21)	325(41.72)	reference	reference	reference	reference	reference
Q2	802(24.92)	280(34.91)	0.749(0.611-0.918)	0.777(0.632-0.955)	0.775(0.629-0.954)	0.782(0.634-0.966)	0.784(0.630-0.973)
Q3	825(25.64)	312(37.82)	0.850(0.695-1.038)	0.859(0.700-1.053)	0.868(0.706-1.066)	0.875(0.711-1.077)	0.871(0.703-1.080)
Q4	812(25.23)	305(37.56)	0.840(0.687-1.028)	0.873(0.711-1.071)	0.881(0.717-1.083)	0.898(0.729-1.106)	0.907(0.731-1.124)
P for trend			0.304	0.463	0.548	0.734	0.818

[a] model 1：Adjusted for the age, plus education level, current smoking status, current alcohol drinking status and physical activity.

[b] model 2：Adjusted for the variables in the model 1 plus coronary heart disease, diabetes, hypertension disease, tumor history and lipid lowering agent.

[c] model 3：Furthered adjusted for the same set of variables in the model 2 plus waist circumference, body mass index, glucose, high-density lipoprotein, total cholesterol, triglyceride and uric acid.

The *ORs* and 95% *CIs* were calculated by unconditional logistic regression after adjusting for above potential confounders. The quartiles of bilirubin levels were calculated by sex respectively. The cutoff values of bilirubin quartiles for males were direct bilirubin (<3.7, 3.7–4.5, 4.5–5.6, and >=5.6 umol/L), indirect bilirubin (<8.0, 8.0–10.6, 10.6–13.6, and >=13.6 umol/L), and total bilirubin (<12.0, 12.0–15.2, 15.2–19.0, and >=19.0 umol/L) respectively, and for females were direct bilirubin (<2.9, 2.9–3.6, 3.6–4.5, and >=4.5 umol/L), indirect bilirubin (<6.6, 6.6–8.6, 8.6–11.2, and >=11.2 umol/L), and total bilirubin (<9.9, 9.9–12.0, 12.0–15.3, and >=15.3 umol/L) respectively.

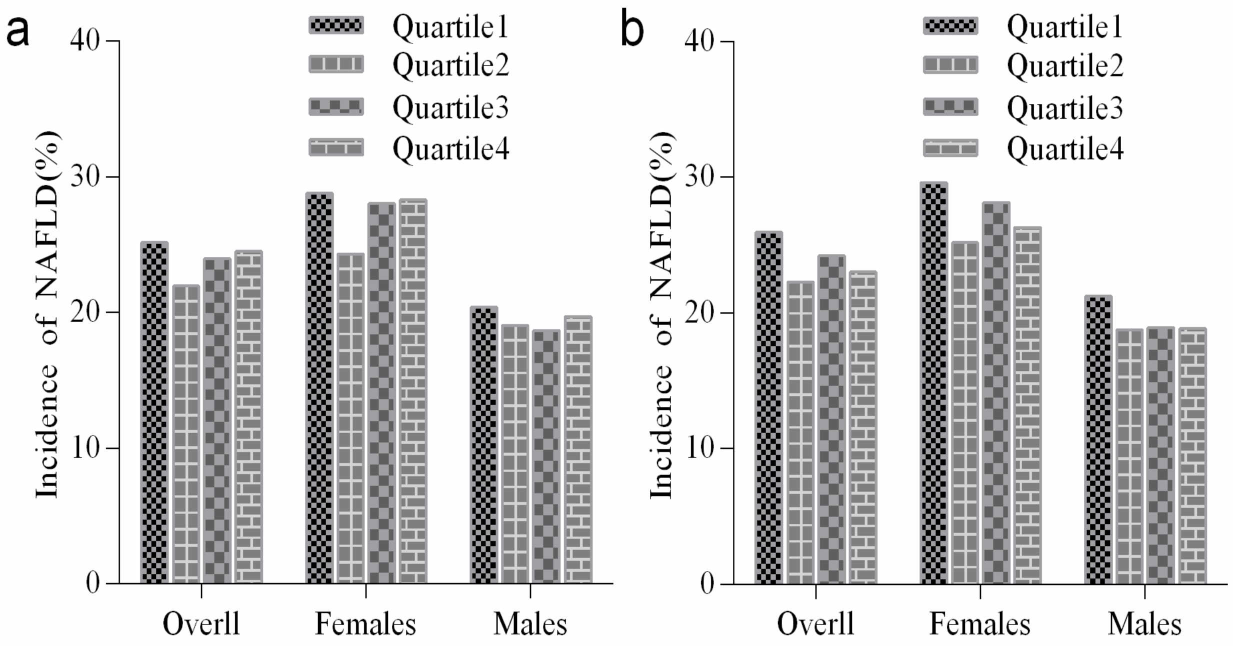

Supplementary figure S 1. The incidence rates of NAFLD according to quartiles of the serum indirect bilirubin (a) and total bilirubin levels (b) in study participants. The quartiles of serum bilirubin levels were calculated by sex respectively. The cutoff values of serum bilirubin levels quartiles for males were, indirect bilirubin (<8.0, 8.0–10.6, 10.6–13.6, and >=13.6 umol/L), total bilirubin (<12.0, 12.0–15.2, 115.2–19.0, and >=19.0 umol/L) respectively, and for females were indirect bilirubin (<6.6, 6.6–8.6, 8.6–11.2, and >=11.2 umol/L), total bilirubin (<9.9, 9.9–12.0, 12.0–15.3, and >=15.3 umol/L) respectively.

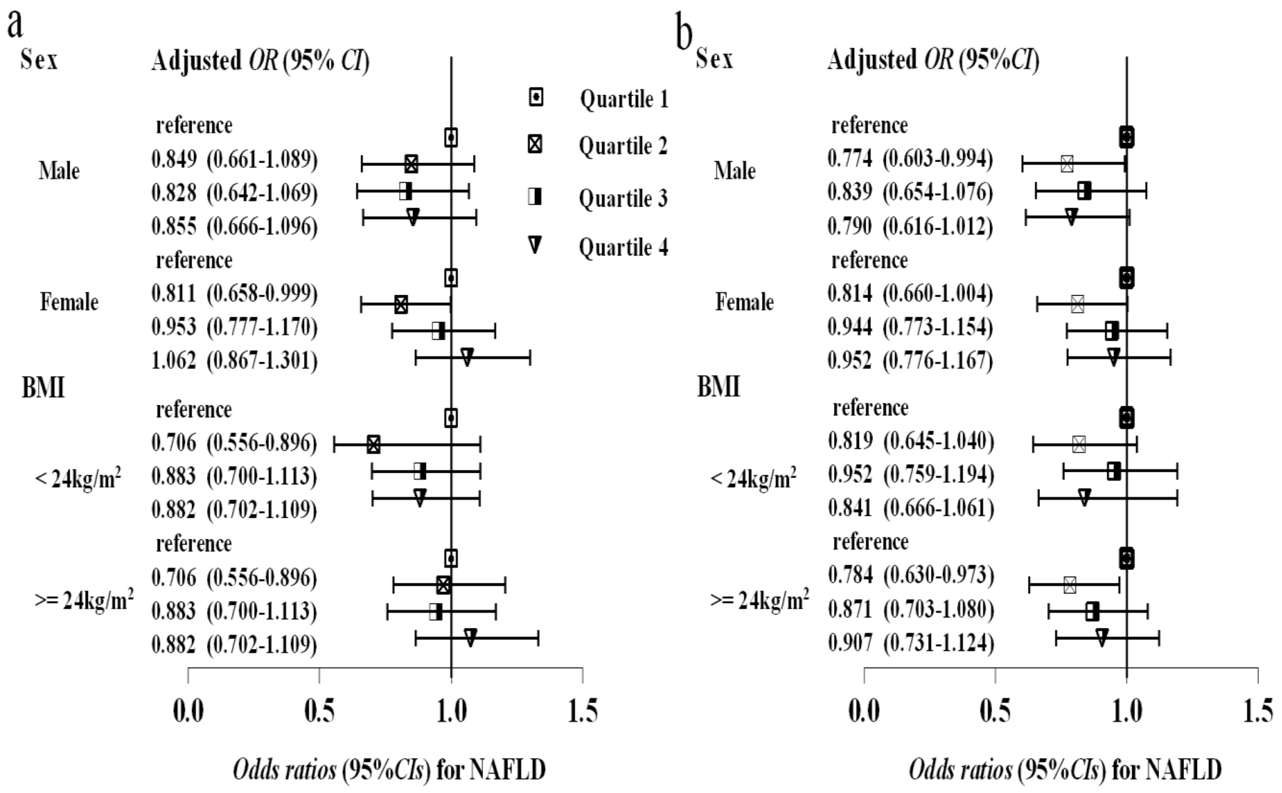

Supplementary figure S 2. Multivariable-adjusted Odds ratios (95% *CIs*) for NAFLD based on serum indirect (a) and total bilirubin levels quartiles (b) respectively stratified by sex and BMI. The *ORs* (95% *CIs*) were presented compared with the quartile 1 of serum bilirubin level (reference), after adjustment for underlying confounders including age, education level, current smoking status, current alcohol drinking status, physical activity, coronary heart disease, diabetes, hypertension disease, tumor history, lipid lowering agent, waist circumference, body mass index, glucose, high-density lipoprotein, total cholesterol, triglyceride and uric acid.
